# The association between social capital and diabetes control: a systematic review and meta-analysis

**DOI:** 10.3389/fpubh.2026.1763272

**Published:** 2026-04-10

**Authors:** Yangyang Chen, Zefeng Chen, Zhan Gu, Jiashu Hu

**Affiliations:** 1 Faculty of Sports Science, Ningbo University, Ningbo, China; 2 Hangzhou Hospital of Traditional Chinese Medicine, Hangzhou, China; 3 Department of Integrative Medicine, Shanghai Pulmonary Hospital, Shanghai, China; 4 School of Economics and Management, Shanghai University of Sport, Shanghai, China

**Keywords:** cognitive social capital, diabetes mellitus, outcome-based health problems, self-care behaviors, structural social capital

## Abstract

**Introduction:**

Diabetes is one of the fastest-growing global health challenges of the 21st century, with estimates suggesting 589 million adults aged 20–79 were living with diabetes in 2024. Although social capital has been shown to be associated with diabetes control, attempts to consolidate the evidence in the form of a systematic review have been limited. This comprehensive systematic review identified and synthesised international research findings of association between social capital and diabetes control to provide a consolidated evidence base to inform future research and policy development.

**Methods:**

All empirical quantitative and mixed methods studies investigating the association between social capital and DM were screened and included from the databases of ELSEVIER ScienceDirect, PubMed and Scopus. Identified literature was screened using review-specific inclusion criteria, the data were extracted from the included studies. The mixed effect sizes were calculated using a random-effects model, then subgroup analyses were conducted to investigate potential sources of heterogeneity.

**Results:**

After screening, 14 studies were enrolled. The findings demonstrated that social capital exerted a statistically significant, albeit small, effect on diabetes control. Social capital exhibited a more robust effect on diabetes control when the effect size was measured using SMD, self-control was measured as a discrete variable and from a behavioral perspective.

**Discussion:**

To date, this is the most comprehensive systematic review and meta-analysis to focus on the association between social capital and diabetes control. It suggests a positive association between social capital and DM control despite various measurements. In addition, it highlights key gaps in knowledge where future research could further illuminate the mechanisms through which social capital works to effect diabetes control and thus inform policy development.

**Systematic review registration:**

https://www.crd.york.ac.uk/PROSPERO/view/CRD42023461071, identifier PROSPERO (CRD42023461071).

## 1 Introduction

Diabetes mellitus stands as the most prevalent metabolic disorder worldwide, exhibiting a dramatic surge in incidence. In 2024, the global population of individuals aged 20–79 years with diabetes reached 589 million, with projections indicating this figure will exceed 853 million by 2050. Diabetes represents a major driver of global mortality, accounting for approximately 3.4 million deaths among adults aged 20–79 years in 2024 due to diabetes or its complications. The condition imposes a substantial economic burden on nations, healthcare systems, individuals with diabetes, and their families. Global health expenditures attributable to diabetes have experienced a significant escalation, rising from 232 billion in 2007 to more than 1 trillion in 2024 (1). Effective prevention and control of diabetes has transcended the realm of a simple health issue, evolving into a significant societal challenge. The development of potential and intangible resources to compensate for material resource deficiencies has become an urgent priority.

In recent years, we have witnessed introduction of the term ‘social capital’ in the realm of science, social sciences, economics, and health sciences. With growing recognition of the social determinants of health, social capital is an increasingly important construct in healthcare (2). Bourdier seemed to be the first to dedicate an entire work to the concept, while further refinements came from Coleman, Putnam, Leonardi, Nanetti, Portes and others (3). Reflecting their disciplinary backgrounds, each of these theorists has conceptualised social capital differently and this has generated debate in the literature about how social capital should be defined and measured. Bourdieu (4) defines social capital in terms of networks and connections between individuals that can provide support and resource, Coleman (5) conceptualises social capital as being a resource of the social relations that exist between families and the communities that they are linked to, and Putnam (6) defines social capital as ‘features of social organization, such as trust, norms and networks, which can improve the efficacy of society by facilitating coordinated actions’. Kawachi et al. (7) have sought a more pluralistic approach that attempts to unify key elements that emerge from the various traditions. This has resulted in relative consensus that social capital includes those elements of social networks that can bring about positive social, economic and health development and this can occur at the micro (individual, family/household) and macro (local, national and international) level.

There are enough consensuses to draw some important generalizations about the nature of social capital (8). The behavioral manifestations of civic engagement or members participation can be classified as structural social capital, and those subjective attitudes (interpersonal trust and norms of reciprocity) as cognitive social capital (9). Despite, or perhaps because of, its complex nature, social capital has been discussed and debated in the public health field by those wishing to explain, reduce and prevent health inequalities (10, 11). Social capital affects human health through the following approaches: diffusing knowledge on health promotion, maintaining healthy behavior, accessing health care services, acquiring emotional or material support, enhancing self-esteem, maintaining mutual respect in social networks, and establishing of a cooperative network among various functional departments of society to promote the efficient allocation and utilization of resources as well as to reduce transaction costs (12).

Although studies have shown that social capital is closely related to diabetes, the precise nature of this association remains debated, that is (1) social capital has significant positive association with diabetes control; (2) social capital has significant negative association with diabetes control; (3) social capital has insignificant correlation with diabetes control; (4) social capital has mixed effect on diabetes control. A small number of reviews do exist but their contribution to the field is limited. For example, Flôr et al. (13) undertook a review to evaluated the association between social capital and control of DM. However, only three papers were included and qualitative analysis was conducted. Hu et al. (14) presented a systematic review to investigate the association between social capital and NCDs (CVD, cancers, COPD, and diabetes), among which 7 studies examined diabetes and meta-analysis was not applied.

In light of limited review-level evidence this current systematic review aims at: (a) identify, analyse and synthesise primary evidence on the association between social capital and diabetes control, (b) make recommendations/discuss implications for future research and policy development. To the best of our knowledge, this is the first comprehensive systematic review and meta-analysis to focus on the association between social capital and diabetes control.

## 2 Methods

### 2.1 Protocol

This systematic review was performed according to the Preferred Reporting Items for Systematic Reviews and Meta-Analysis guidelines (PRISMA) (15). The eligibility and exclusion criteria and the search strategy were made and agreed upon by two authors (Yangyang Chen and Zefeng Chen) with *a priori* to minimize bias. The PROSPERO Registration Number of this systematic review was CRD42023461071.

### 2.2 Criteria for inclusion

#### 2.2.1 Types of studies

Facilitated by our integrative approach we sought to include primary empirical quantitative and mixed methods studies that were published and peer reviewed. Literature review, revisions, letters to the editor, comments to thesis, or abstracts published in annals of congress were excluded. Studies were also excluded if non-English published.

#### 2.2.2 Types of participants

Individuals diagnosed with diabetes mellitus, with no geographical restrictions or age restrictions applied.

#### 2.2.3 Types of social capital

While there is no ‘set’ definition of social capital in use, this paper understands social capital to refer to the degree of connectedness and the quality and quantity of social relations in a given population. Bain and Hicks (9) disaggregates social capital into two components: structure and cognitive. The structure component refers to the extent and intensity of associational links or activity, was operationalized to include the following constructs: membership, social integration (e.g., participation in community organizations or clubs), collective action and cooperation, empowerment and political action, and access to specialized professionals. The cognitive component covers perceptions of support, reciprocity, sharing and trust, typically further detailed as trust (trust and solidarity, generalized trust, neighborhood trust), social support (emotional, informational, and practical support), along with social cohesion and inclusion. At the simplest level, these two components can be, respectively, characterized as what people ‘do’ and what people ‘feel’ in terms of social relations. Therefore, only studies that included an indicator of structure and/or cognitive social capital were considered for inclusion. Only studies that conceptualised and/or measured social capital as predicting, or influencing the improvement in the control of DM were considered for inclusion; studies were not included if they conceptualised social capital as an outcome variable.

#### 2.2.4 Types of outcomes

Studies were included if they assessed individual-level self-care behaviors and/or health problems. Self-care behaviors were operationalized by: diabetes control, self-attainment of diet and exercise, weight management and self-reported medication adherence. In contrast, health problems are outcome-based: HbA1c, blood pressure and low-density lipoprotein cholesterol level, complications (neuropathy, retinopathy, nephropathy, cardiovascular disease, diabetes duration and depression). Only studies that conceptualised and/or measured self-care behaviors and/or health problems as outcome variables were considered for inclusion.

### 2.3 Search strategy

In order to identify all empirical quantitative and mixed methods studies investigating the association between social capital and DM published up to 31th July 2025, a comprehensive reproducible search strategy was performed on the databases of ELSEVIER ScienceDirect, PubMed and Scopus. The search terms used in each database were as follows: (1) in ELSEVIER ScienceDirect, the search term was tak((“Diabetes Mellitus” OR “diabetes” OR “T2DM”) AND (“social capital” OR “social support” OR “social networks” OR “social cohesion” OR “neighborhood factors”) AND NOT (“qualitative” OR “qualitative study”)); (2) in PubMed, the search term was (“Diabetes Mellitus”[Mesh] OR “diabetes”[Title/Abstract] OR “T2DM”[Title/Abstract]) AND (“social capital”[Title/Abstract] OR “social support”[Title/Abstract] OR “social network*”[Title/Abstract] OR “social cohesion”[Title/Abstract] OR “community cohesion”[Title/Abstract] OR “collective efficacy”[Title/Abstract] OR “neighborhood factors”[Title/Abstract]) NOT (“qualitative”[Title/Abstract] OR “qualitative study”[Title/Abstract]); (3)in Scopus, the search term was TITLE-ABS-KEY((“Diabetes Mellitus” OR “diabetes” OR “T2DM”) AND (“social capital” OR “social support” OR “social network*” OR “social cohesion” OR “neighborhood factors”) AND NOT (“qualitative” OR “qualitative study”)).

### 2.4 Study selection

All potential studies were imported into EndNote X9 and duplicates were removed. Title, abstract, and full-text screening was conducted independently by two authors (Yangyang Chen and Zefeng Chen). Any disagreement would be resolved by a third independent reviewer (Jiashu HU).

### 2.5 Data extraction

All searched studies were imported into EXCEL for further screening. The following information would be collected and recorded: author, year of publication, setting, study design, sample size, patients age, gender, instrument adopted to measure social capital, type of social capital measured (cognitive and/or structural), a parameter to evaluate DM, and main results. Quality appraisal was carried out at the same time as data extraction. The two authors used a study-specific quality appraisal tool (QAT), which was developed to enable appraisal of studies with a range of research designs. The QAT included 11 criteria covering: whether the theoretical framework underpinning the research was explicitly described; explicit reporting of the study aims and objectives; the concordance between the stated aims and the methodological approach; the rigour and reporting of the results; and, the appropriateness of the conclusions drawn. Criteria were scored on a three-point scale (0 = weak, 1 = moderate, 2 = strong), giving a possible range of scores from 0 to 22 for each study. Disagreements were resolved through discussion, involving a third author if necessary. Each study was then awarded a quality rating: studies scoring between 16 and 22 were awarded a ‘high quality’ rating; studies scoring between 8 and 15 were awarded a ‘moderate quality’ rating; and, studies scoring between zero and seven were awarded a ‘low quality’ rating. We did not exclude studies on the grounds of quality.

### 2.6 Meta-analysis

Following data extraction, the standardized mean difference (SMD) was calculated using Hedges’ *g*, a bias-corrected version of Cohen’s *d* (16), to normalize effect sizes across studies for subsequent analyses. Hedges’ *g* values were computed directly in R 4.1.2 by inputting the sample sizes, baseline and post-intervention means, and standard deviations of the study populations. In cases where means or standard deviations were unavailable, these values were estimated from available test statistics including *t*-values, *F*-values, or standard errors. The magnitude of effect sizes was interpreted according to conventional thresholds: 0.2 representing a small effect, 0.5 a medium effect, and 0.8 a large effect (17).

To address statistical dependency arising from multiple effect sizes within studies, we used robust variance estimation (RVE) with random-effects models. RVE adjusts standard errors for correlated effects without requiring knowledge of the exact covariance structure. We assumed a within-study correlation of *ρ* = 0.8 in the primary analysis and conducted sensitivity analyses with *ρ* = 0.4, results were consistent across values.

The mixed effect sizes were calculated using a random-effects model, which accounts for potential heterogeneity across studies. Concurrently, heterogeneity among effect sizes was evaluated using Cochran’s *Q* test and the *I*^2^ statistic. The *I*^2^ metric quantifies the proportion of total variance attributable to between-study variation, with thresholds of 25, 50, and 75% indicating low, medium, and high heterogeneity, respectively. A significant *Q* test accompanied by *I*^2^ ≥ 75% suggests substantial heterogeneity, justifying the use of a random-effects model (18). In such cases, further investigations—including subgroup analyses and moderator analyses—are warranted to identify sources of heterogeneity and factors significantly influencing effect sizes.

### 2.7 Sensitivity analysis

The selection of literature inclusion criteria, data extraction methods, and approaches to handling missing values can significantly influence the outcomes of a meta-analysis. Therefore, conducting sensitivity analyses to evaluate the impact of these critical factors is essential (19). In this study, robustness was assessed by identifying and excluding outliers—defined as studies whose reported effect sizes exhibited 95% confidence intervals that did not overlap with the 95% confidence interval of the pooled effect size. Such studies were classified as non-contributory and removed. The effect sizes were then recalculated to verify the stability of the results. This approach ensures methodological rigor and enhances the reliability of the meta-analytic conclusions.

### 2.8 Subgroup analyses and meta-regression

To investigate potential sources of heterogeneity, this study employed a mixed-effects model to conduct subgroup analyses and meta-regression. Initially, subgroup analyses were performed for each categorical variable to assess its independent influence on intervention effects. While this approach identifies broad patterns, it does not account for potential correlations or interactions between variables (20). To address this limitation, meta-regression was subsequently applied to examine moderating factors and their interactions more rigorously. Specifically, subgroup analyses were restricted to variables with at least four studies per group to ensure statistical reliability. For meta-regression, only factors represented by ten or more studies were included.

Guided by the principles of evidence-based medicine, this study systematically examines potential determinants of diabetes control through the Population, Intervention, Comparison, and Outcome (PICO) framework (21), integrating insights from existing literature. The key factors are categorized as follows: (1) Population Characteristics: demographic variables, including sex distribution and age; (2) Intervention Characteristics: Measure Type of social capital; (3) Research Design: Research Type (e.g., SMD-based, *Z*-scores-based); (4) Outcome Measures: Measure Type of Diabetes Self-Control, measure view of Diabetes Self-Control.

### 2.9 Risk of bias

Studies reporting statistically significant results are more likely to be published, a phenomenon known as publication bias. This study employed multiple approaches to evaluate publication bias potentially induced by small-study effects. These included enhanced funnel plots for visual asymmetry assessment and Egger’s linear regression test for quantitative bias detection. Subsequently, Duval & Tweedie’s Trim-and-fill Procedure and PET-PEESE methods were applied to recalculate adjusted effect sizes. The robustness of findings was determined by comparing pre- and post-adjustment effect magnitudes, where minimal divergence indicates limited publication bias influence. This study employed Cohen’s kappa coefficient to evaluate inter-rater reliability in coding consistency. The kappa values were interpreted according to established thresholds: 0.40–0.59 indicated good agreement, 0.60–0.74 represented substantial agreement, and values ≥0.75 denoted excellent agreement (22).

## 3 Results

### 3.1 Search strategy and data extraction

**Figure 1:**
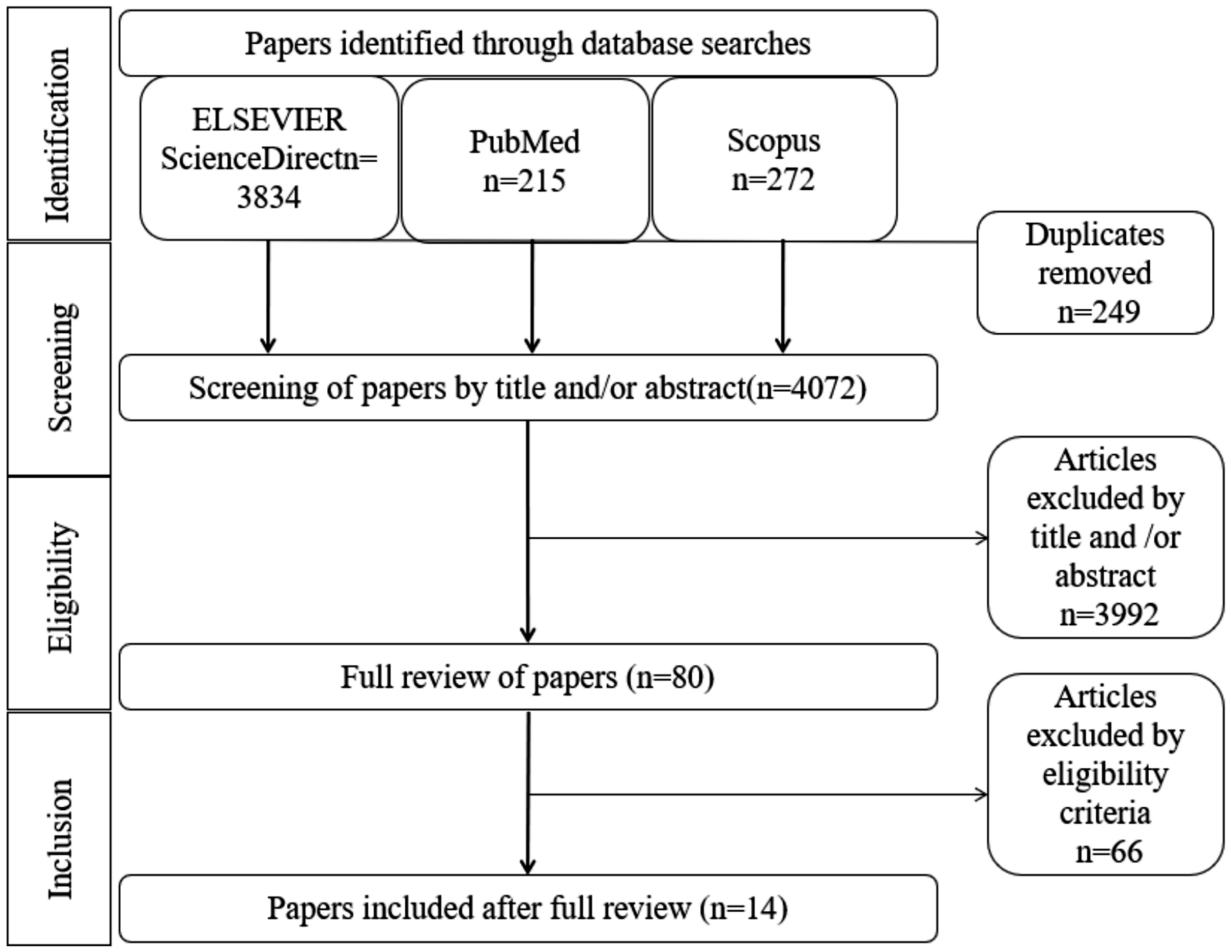
Procedure of studies selection.

A total of 14 studies were enrolled in the final analysis (23–36). Figure 1 shows the procedure of studies selection. The information of all included studies is presented in Table 1.

**Table 1:** Characteristics of 14 studies measuring social capital and DM control.

Study	Setting	Sample size	Age (years)	Gender (male/female)	Social capital measure	Diabetes measure
Long et al., (23)	USA	294	≥29	Male	Participate, community rate, help, improve, belong, trust	HbA1c
Farajzadegan et al. (24)	Iran	120	≥30	22/98	Groups and networks, trust and solidarity, collective action and co-operation, information and communication, social cohesion and inclusion, empowerment and political action	HbA1c
Riumallo-Herl et al. (25)	Chile	4,209	30–100	1685/2524	Social support (emotional), generalized trust (society as a whole, thin trust) and neighborhood trust(thick trust)	Self-rated health, measured blood pressure and assessed blood sugar
de Vries McClintock et al. (26)	USA	180	≥30	59/121	Social affluence, residential stability, neighborhood advantage	Diabetes Control (patterns of adherence to oral hypoglycemic agents)
Smalls et al. (27)	USA	615	≥18	379/236	Social cohesion, social support	HbA1c, blood pressure and low-density lipoprotein cholesterol level, self-reported medication adherence, self-care behaviors
Koetsenruijter et al. (28)	6 European countries	1,692	66.2	846/846	Individual support networks (information support, practical support, emotional support), community organisations, access to specified professionals	Self-management capabilities
Mendoza-Núñez et al. (29)	Mexico	182	≥60	29/153	Familial, extra-familial, institutional	HbA1c
Mizuno et al. (30)	Japan	8,874	31.04 ± 5	Female	Social support, neighborhood trust, subjective local security, trust in people	HbA1c
Waverijn et al. (31)	Netherlands	2091	16–93	899/1192	Neighbourhood social capital	Self-rated health, self-management
Moradi et al. (32)	Iran	300	43.05 ± 6.8	88/212	Groups and networks, trust and solidarity, collective action and cooperation, information and communication, social cohesion and inclusion, empowerment and political action	HbA1c
Kamimura et al. (33)	USA	374	46.01 ± 14.28	120/254	Membership, influence, reinforcement of needs, shared emotional connection	Physical activity adherence, weight management, and low-salt diet adherence
Yamada et al. (34)	Japan	65	43–85	42/23	Trust in people in a community, social support, social relationships	HbA1c, self-attainment of diet (SAD), self-attainment of exercise (SAE), complications
McEwen et al. (35)	USA	157	53.53 ± 9	55/102	Social integration, social support, collective efficacy,	HbA1c
Sanjari et al. (36)	Iran	435	28–71	Female	Feeling of trust and safety in local community, feeling of trust for formal participation in the community, feeling of safety at night in the community, tolerance of diversity and social participation, neighborhood connections, value of life, family and friends connections, proactivity in the social context, social work	HbA1c, diabetes control, self-rated health status, depression

### 3.2 Meta-analysis

The heterogeneity testing was conducted on the included effect sizes to determine the appropriateness of employing a random-effects model and the necessity for subsequent moderator analyses. Table 2 revealed significant heterogeneity [*Q*_(115)_ = 188.29, *p* < 0.001] for Diabetes Self-Control, with an *I*^2^ statistic of 38.9%, indicating moderate heterogeneity. Although the *I*^2^ value suggests a relatively low proportion of between-study variance, the statistically significant *Q* test implies that a non-negligible fraction of the observed variability stems from true differences in effect magnitudes. Consequently, a random-effects model was selected to account for this underlying heterogeneity in the account for this underlying heterogeneity in the pooled analysis. Thus, moderator analyses are warranted to identify potential sources of variation and refine the interpretation of the aggregated results. The findings of these subgroup and moderator analyses are presented below to explore the observed heterogeneity.

**Table 2:** Heterogeneity, the pooled effect size and sensitivity analysis.

Variable	*k*	Heterogeneity			The pooled effect size		Sensitivity analysis	
		*Q*	*I* ^2^	*τ* ^2^	*g* (95% CI)	Prediction 95% CI	*g* (95% CI)	Prediction 95% CI
Diabetes Self- Control	116	*Q*(115) = 188.29, *p* < 0.001	38.9%	0.014	0.062*** [0.037; 0.087]	[−0.173, 0.297]	0.049*** [0.027, 0.072]	[−0.161, 0.259]

***p < 0.001; **p< 0.01; *p < 0.05.

The pooled effect size for Diabetes Self-Control was estimated using a random-effects model, yielding a statistically significant point estimate of 0.062 (95% CI: 0.037 to 0.087), with the confidence interval excluding null. In accordance with the classification proposed by Kallapiran et al. (17), this magnitude corresponds to a small effect size, suggesting a modest but consistent positive association between social capital and Diabetes Self-Control.

### 3.3 Sensitivity analysis

Following the identification and exclusion of outliers, Table 2 shows the pooled effect size estimate for Diabetes Self-Control was 0.049 (95% CI: 0.027 to 0.072), with the confidence interval excluding null. Compared to the pre-exclusion estimate, the point estimate decreased by 0.013, and the upper confidence limit reduced by 0.015, indicating minimal divergence. These results collectively demonstrate high stability in the meta-analytic conclusions, reinforcing the reliability of the observed small but statistically significant effect.

### 3.4 Subgroup analyses

To explore potential sources of heterogeneity and test theoretically driven hypotheses, we conducted subgroup analyses based on the following moderators, selected for both conceptual and methodological reasons: (1) Research Type: Study design (e.g., cross-sectional vs. experimental) may influence effect size estimates due to differences in causal inference strength, recall bias, and temporal relationships. Examining this moderator helps assess whether the observed associations are robust across different study designs; (2) Measure Type of Social Capital (Cognitive vs. Structural): Social capital theory distinguishes between cognitive and structural dimensions, which are hypothesized to operate through different pathways—psychological versus behavioral and instrumental. This subgroup analysis tests whether these theoretical distinctions translate into differential effect sizes; (3) Measure Type of Diabetes Self-Control (Continuous vs. Discrete): Continuous measures typically capture perceived capabilities or propensities, while discrete measures capture behavioral attainment. Given the well-documented ‘intention-behavior gap’ in health psychology, social capital—particularly through social norms and social support—may exert stronger effects on discrete, socially observable behaviors. This analysis examines that hypothesis; (4) Measure Perspective of Diabetes Self-Control (Behavioral vs. Outcome-based): Behavioral measures reflect the process of self-management, whereas outcome-based measures reflect physiological endpoints. Social capital may influence these two types of outcomes through different mechanisms and with different time lags, warranting separate examination; (5) Measure Perspective of Diabetes Self-Control (Mental vs. Physical): Mental health indicators (e.g., depression) and physical health indicators are interrelated but distinct aspects of diabetes self-Control. Exploring whether social capital differentially affects these dimensions can inform integrated care models. Table 3 revealed statistically significant variation in effect sizes across Research Type [*Q*_(1)_ = 4.28, *p* = 0.0385], indicating that the magnitude of Diabetes Self-Control effects differed meaningfully between subgroups. Specifically, when the effect size was measured using SMD, the pooled effect size estimate for Diabetes Self-Control was 0.086 (95% CI: 0.048 to 0.123), with the confidence interval excluding null and exhibiting heterogeneity. When the effect size was measured using *Z*-scores, the pooled effect size estimate for Diabetes Self-Control was 0.035 (95% CI: 0.005 to 0.066), with the confidence interval excluding null.

**Table 3:** Subgroup analyses of Research Type (SMD vs. *Z*-scores).

Variable	Variation	Regression				
	*Q*	Research Type	*k*	*g* (95% CI)	*Q*	*I* ^2^
Diabetes Self-Control	*Q*_(1)_= 4.28, *p =* 0.0385	SMD	63	0.086 [0.048, 0.123]	119.48	48.1%
		Zscore	53	0.035 [0.005, 0.066]	63.03	17.5%

***p < 0.001; **p< 0.01; *p < 0.05.

Table 4 revealed no statistically significant variation in effect sizes across Measure Type of Social Capital [*Q*_(1)_ = 0.001, *p* = 0.9939], indicating that the magnitude of Diabetes Self-Control effects did not differ meaningfully between subgroups. This result suggests that the choice of social capital measurement approach (cognitive vs. structural) does not systematically influence the observed effects of Diabetes Self-Control.

**Table 4:** Subgroup analyses of Measure Type of Social Capital (Cognitive vs. Structural).

Variable	Variation	Regression				
	*Q*	Social Capital	*k*	*g* (95% CI)	*Q*	*I* ^2^
Diabetes Self-Control	*Q*_(1)_ = 0.001, *p =* 0.9939	Cognitive	41	0.062 [0.025, 0.100]	95.55	58.1%
		Structural	75	0.062 [0.028, 0.097]	92.16	19.7%

***p < 0.001; **p< 0.01; *p < 0.05.

Table 5 revealed statistically significant variation in effect sizes across Measure Type of Diabetes Self Control [*Q*_(1)_ = 9.02, *p* = 0.0027], indicating that the magnitude of Diabetes Self-Control effects differed meaningfully between subgroups. Specifically, when self-control was measured as a continuous variable, the pooled effect size was 0.031 (95% CI: 0.004 to 0.059), with the interval excluding null. Conversely, when self-control was operationalized as a discrete variable, the effect size estimate was substantially larger at 0.105 (95% CI: 0.064 to 0.147), demonstrating statistical significance and heterogeneity. These results suggest that the methodological choice of self-control measurement (continuous vs. discrete) systematically influences the observed effects of Diabetes Self-Control. Table 5 also showed statistically significant variation in effect sizes across Measure Perspective of Diabetes Self-Control [*Q*_(1)_ = 4.93, *p* = 0.0264], indicating that the magnitude of Diabetes Self-Control effects differed meaningfully between subgroups. Specifically, when self-control was measured from a behavioral perspective, the pooled effect size was 0.092 (95% CI: 0.055 to 0.127), with the interval excluding null and exhibiting heterogeneity. Conversely, when self-control was operationalized from an outcome-based perspective, the effect size estimate was 0.037 (95% CI: 0.003 to 0.070), also demonstrating statistical significance and heterogeneity. These results suggest that the methodological choice of self-control measurement perspective (behavioral vs. outcome-based) systematically influences the observed effects of Diabetes Self-Control. Regarding another measure perspective (mental vs. physical) of self-control, no significant subgroup differences were found for the effect of Diabetes Self-Control [*Q*_(1)_ = 1.23, *p* = 0.267].

**Table 5:** Subgroup analyses of Measure Type & Perspective of Diabetes Self Control.

Variable	Variation	Regression				
	*Q*	Diabetes Self-Control	*k*	*g* (95% CI)	*Q*	*I* ^2^
Diabetes Self-Control	*Q*_(1)_ = 9.02, *p =* 0.0027	Continuous	67	0.031 [0.004, 0.059]	69.17	4.6%
		Discrete	49	0.105 [0.064, 0.147]	102.67	53.2%
	*Q*_(1)_ = 4.93, *p =* 0.0264	Behavioral	52	0.092 [0.055, 0.127]	79.49	35.8%
		Outcome-based	64	0.037 [0.003, 0.070]	108.50	41.9%
	*Q*_(1)_= 1.23, *p* = 0.2666	Mental	14	0.102 [0.019, 0.185]	28.08	53.7%
		Physical	102	0.057 [0.031, 0.083]	155.35	35.0%

***p < 0.001; **p < 0.01; *p < 0.05.

### 3.5 Meta-regression

To identify the most influential predictors of the effect size for Diabetes Self-Control, a Multi-model Inference Analysis (37) was performed, integrating factors such as Measure Type of Social Capital, Measure Type of Diabetes Self-Control and Measure Perspective of Diabetes Self-Control. The meta-regression analysis (Table 6) revealed that only the Measure Type of Diabetes Self-Control was a significant moderator. In terms of relative importance, the Research Type was the foremost factor, succeeded by age, Measure Type of Social Capital, gender, the mental/physical perspective of Diabetes Self-Control, and the behavioral/outcome-based perspective of Diabetes Self-Control.

**Table 6:** Results of meta-regression.

Variable	Diabetes Self Control	
	*B* (SE)	Importance
Age	0.005 (0.005)	0.9889
Gender (Ref: male)		0.7751
Female	−0.002 (0.001)	
Measure Type of Diabetes Self-Control (Ref: continuous)		0.9998
Discrete	0.412*** (0.094)	
Measure perspective of Diabetes Self-Control (Ref: mental)		0.5250
Physical	−0.079 (0.106)	
Measure perspective of Diabetes Self-Control (Ref: behavioral)		0.2994
Outcome-based	0.003 (0.055)	
Measure Type of Social Capital (Ref: structural)		0.7875
Cognitive	0.131 (0.096)	

***p < 0.001; **p < 0.01; *p < 0.05.

### 3.6 Risk of bias assessment

**Figure 2:**
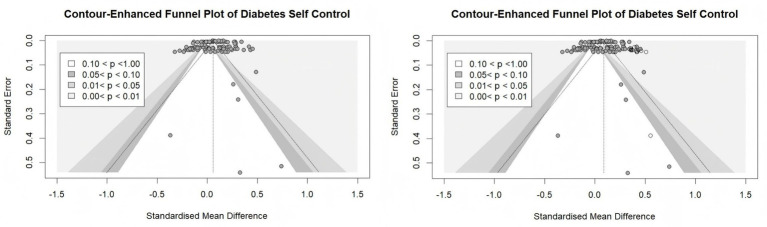
Funnel plot for diabetes self-control before and after trim-and-fill adjustment.

The funnel plot for Diabetes Self-Control (Figure 2) showed that effect sizes were predominantly concentrated in the upper portion of the graph and symmetrically distributed around the pooled effect, which is consistent with the absence of publication bias. This visual impression was supported by Egger’s linear regression test (intercept = −4.006, 95% CI: −8.86 to 0.85, *p* > 0.05), which did not indicate significant asymmetry.

However, other methods suggested potential publication bias. Duval & Tweedie’s Trim-and-Fill procedure (Table 7) imputed missing studies and yielded an adjusted effect size (*g* = 0.089, 95% CI: 0.056 to 0.121) that differed from the unadjusted estimate. Similarly, the PET-PEESE method produced adjusted estimates (PET intercept: 0.079, 95% CI: 0.042 to 0.115; PEESE: 0.066, 95% CI: 0.043 to 0.090) that were also divergent from the primary meta-analysis result.

**Table 7:** Publication bias assessment.

Publication bias assessment			
Egger’s intercept	Trim and Fill *g* (95% CI)	PET’s intercept	PEESE’s intercept
−4.006 [−8.86, 0.85]	0.089*** [0.056, 0.121]	0.079** [0.042, 0.115]	0.066*** [0.043, 0.090]

***p < 0.001; **p < 0.01; *p < 0.05.

These discrepant findings likely reflect the limitations of different publication bias tests in the presence of substantial heterogeneity (*I*^2^ = 38.9%). Egger’s test may have low power to detect asymmetry when between-study variance is high, while Trim & Fill and PET-PEESE can be sensitive to the influence of small studies even when no systematic bias exists.

Given the inconsistency across methods, we cannot definitively conclude the absence of publication bias. Instead, we interpret these findings as indicating that publication bias cannot be ruled out. If present, the direction of the bias would likely be toward overestimation of the pooled effect, as smaller studies with positive findings may be overrepresented. This possibility is acknowledged as a limitation of the current meta-analysis.

## 4 Discussion

The primary aim of this systematic review was to identify, analyse and synthesise empirical evidence on the association between social capital and diabetes control. In doing so we assessed evidence from 14 studies making this the most comprehensive systematic review and meta-analysis in this field.

The findings demonstrated that social capital exerted a statistically significant, albeit small, effect on diabetes control. However, it is important to interpret this overall effect with caution, as our heterogeneity analysis revealed significant between-study variation (*Q* test *p* < 0.001) with a moderate *I*^2^ value of 38.9%. This indicates that the true effects of social capital on diabetes control are not uniform across studies, and the pooled estimate should be viewed as an average of varying effects rather than a single universal truth. The subsequent subgroup and moderator analyses were conducted to explore sources of this heterogeneity and are discussed below. While the pooled effect size of social capital on diabetes control was small, its practical significance should be considered from several perspectives. First, from a population health perspective, even a small effect can have substantial implications when applied at the community or population level. For example, a modest reduction in HbA1c across a large population could translate into meaningful decreases in diabetes-related complications and healthcare utilization. Second, in terms of intervention feasibility and scalability, interventions targeting social capital—such as community trust-building initiatives or peer support programs—are often low-cost, sustainable, and can be integrated into existing community structures. Even a small effect from such interventions may be cost-effective compared to resource-intensive clinical alternatives. Third, the small overall effect size may reflect measurement heterogeneity and the early stage of this research field. Taken together, while the overall effect is modest, it should not be dismissed as clinically irrelevant. Rather, it highlights the potential of social capital as a complementary target for diabetes self-Control, particularly when leveraged through community-based, behaviorally-focused interventions. Future research with more refined measurements and study designs may reveal conditions under which the effect is amplified.

When the effect size was measured using SMD, the observed pooled effect size of social capital on diabetes control was more robust compared to analyses using *Z*-scores. The superiority of SMD stems from its methodological advantages, including universal applicability across studies, statistical robustness, and enhanced clinical interpretability. Specifically, SMD standardizes group differences by the pooled standard deviation, placing results from different measurement instruments (e.g., various HbA1c assays, different self-care scales) onto a common metric. This enables meaningful cross-study comparisons that are essential for meta-analytic synthesis. In contrast, Z-scores are derived from study-specific distributions and lack a uniform scale, making them unsuitable for pooling across studies. Furthermore, Hedges’ *g*—a bias-corrected version of Cohen’s *d*—was used to account for small-sample bias, enhancing the accuracy of individual effect size estimates. The resulting SMD values can be interpreted using conventional benchmarks (small: 0.2, medium: 0.5, large: 0.8), providing intuitive insight into the practical significance of the findings. These properties establish SMD as a reliable metric for this meta-analysis, consistent with the Cochrane Handbook’s recommendation for continuous outcomes.

This study found no significant difference in the effect size of social capital on diabetes control when social capital was measured across cognitive versus structural dimensions. Among the 14 included studies, cognitive social capital was operationalized by indicators including: Trust (trust and solidarity, generalized trust, neighborhood trust), Social support (emotional, informational and practical support), Social cohesion and inclusion. These cognitive components were associated with enhanced intra- and inter-community psychological outcomes, such as improved sense of security and self-esteem. Conversely, structural social capital was measured via: Membership, Social integration (participation in community organizations or clubs), Collective action and cooperation, Empowerment and political action, Access to specialized professionals. The structural dimension facilitates connections to formal and informal institutional resources, which mitigate the adverse effects of life stressors by providing additional support. Drawing on the mechanisms outlined in the Introduction, our findings suggest that social capital influences diabetes self-Control through two primary pathways. First, the psychosocial pathway—encompassing emotional support, self-esteem enhancement, and mutual respect—operates by improving patients’ psychological readiness and motivation for self-care. Second, the behavioral and instrumental pathway—including health knowledge diffusion, healthy behavior maintenance, access to services, and resource allocation—operates by providing tangible tools and social structures that enable effective diabetes self-Control. These pathways are not mutually exclusive but rather work synergistically, as evidenced by our subgroup analyses showing differential effects based on measurement approaches.

These findings carry distinct implications for health system planning. Interventions aimed at enhancing cognitive social capital—such as community-based trust-building initiatives or peer support groups—could be integrated into public health strategies to improve patients’ psychological readiness for self-management. Meanwhile, strengthening structural social capital calls for health system investments in community infrastructure, such as partnering with local organizations to facilitate patient access to specialized professionals or structured group activities, thereby providing tangible resources that buffer life stressors.

The results of this study revealed that social capital exhibited a more robust effect on diabetes control when self-control was measured as a discrete variable. This finding helps explain part of the heterogeneity observed in our main analysis: studies using discrete behavioral measures tend to report larger effects, contributing to the between-study variance detected by the *Q* test. Firstly, continuous variables typically measure underlying capabilities or propensities. For instance, the ‘Self-Monitoring and Insight’ (SMI) scale captures the individual’s perceived ability to monitor their condition and reflect on how self-management actions influence their status (28). However, high intention does not always translate into successful action, illustrating the well-documented ‘intention-behavior gap’. In contrast, discrete variables typically measure behavioral attainment, directly capturing the performance of effective behaviors, which are the most critical outcomes in diabetes control. Secondly, discrete behavioral targets are more susceptible to the influence of social norms. A precisely defined goal, such as “do at least 30 min of physical activity,” is more readily subject to monitoring, evaluation, and enforcement by one’s social network than a continuous, vague psychological state (e.g., ‘I have a very good idea of how to manage my health problems’). When a behavior is clearly defined as ‘good’ or ‘bad’ within a community, social capital can exert a more potent effect through the constraints and incentives of social norms. This has direct implications for public health programming. To leverage the power of social norms, interventions should translate broad goals into specific, observable behavioral targets that communities can collectively monitor and reinforce. For policymakers, evaluation frameworks for diabetes programs should prioritize such discrete behavioral outcomes alongside clinical indicators, as they may be more sensitive to the influence of community-level social capital.

This study also demonstrated that social capital exhibited a more robust effect on diabetes control when diabetes self-control was assessed from a behavioral perspective compared to outcome-based indicators. The stronger effect observed for behavioral outcomes suggests that the type of outcome measure is a significant source of heterogeneity, reinforcing the need for careful consideration of outcome selection in future research. While both measurement approaches validly reflect diabetes self-control, self-care behavioral metrics uniquely capture self-efficacy—a critical psychological determinant of sustained diabetes control.

This study revealed no significant difference in the effect size of social capital on diabetes self-control, regardless of whether diabetes self-control was measured from a mental or physical perspective. Firstly, the analysis was substantially underpowered to detect a true difference, as the mental subgroup was represented by only 2 studies (assessing depression, self-attainment), compared to 14 studies utilizing physical metrics (e.g., HbA1c, blood pressure). While the pooled effect size did not reach statistical significance, this finding should be interpreted with caution due to limited statistical power. It is possible that a true effect exists but could not be detected with the available data. Future research with more studies examining mental health outcomes is needed to draw firm conclusions. Secondly, the observed lack of differentiation may be attributable to the inherent covariation between mental and physical dimensions. For instance, emotional stress can directly dysregulate blood glucose levels, while effective glycemic control can enhance mental well-being. This interdependence suggests that social capital may exert its influence through shared, unmeasured pathways that concurrently benefit both dimensions, resulting in similar effect sizes across subgroups. The observed covariation between mental and physical dimensions underscores the need for integrated care models. Health system planners should consider embedding mental health support within routine diabetes care at the community level, as interventions that leverage social capital are likely to yield synergistic benefits for both psychological well-being and glycemic control.

The findings of this study align with those of Flôr et al. (13). However, they diverge from Hu et al. (14), who reported mixed results: four of six estimates showed a significant inverse association between cognitive social capital and diabetes, while one study found a significant positive association. For structural social capital, most studies in Hu et al. found no association, although one reported a significant inverse association. Social capital is context-dependent, and findings from one setting may not be directly transferable to another. In contrast to the aforementioned systematic reviews, this study conducted extensive subgroup analyses, which revealed that while the overall effect size of social capital on diabetes control was small, specific conditions significantly enhanced its efficacy.

However, there are several limitations of this study. First, in terms of the individual studies, social capital is a multifaceted concept whose dimensions function in various directions, the lack of an agreed definition and little uniformity in its measurement across the studies made synthesising the evidence challenging. To address this, we employed well-defined study-specific inclusion/exclusion criteria to ensure relevant data were captured in an objective fashion. A more consistent and robust approach to defining and operationalizing social capital in future research would be beneficial, thereby clarifying its relationship with important outcomes and supporting more reliable inter-study comparisons. Second, the majority of included studies employed cross-sectional designs, which precludes any inference about causality or the temporal direction of the observed associations. While our findings demonstrate a significant association between social capital and diabetes control, reverse causality cannot be ruled out: it is possible that individuals with poorer diabetes control experience reduced social participation and networks, leading to lower social capital. Longitudinal studies are needed to establish temporal sequences and causal pathways. Third, despite our subgroup analyses exploring different dimensions and measurements of social capital, substantial measurement variability remains. Studies used diverse instruments and indicators to capture social capital, which may introduce heterogeneity and limit the comparability of findings. Future research should work toward standardized, validated measures of social capital in the context of chronic disease management. Fourth, as with all observational research, residual confounding is a potential concern. Although the included studies adjusted for various covariates, unmeasured or imperfectly measured confounders—such as socioeconomic status, health literacy, comorbid conditions, or access to healthcare—may have influenced the observed effect sizes. This could lead to overestimation or underestimation of the true association. Fifth, most of the 14 articles measure social capital at the individual level, more studies are needed to understand the whether the association is the same for the “macro” level (social, political and economic background) and the “meso” level (neighborhood or community), as social capital may operate differently across levels. Sixth, our assessment of publication bias yielded mixed results. While Egger’s test and visual inspection of the funnel plot did not indicate significant asymmetry, the Trim & Fill and PET-PEESE methods suggested potential bias. Given the heterogeneity among included studies, these discrepant findings should be interpreted cautiously. We cannot exclude the possibility that our pooled effect size is slightly overestimated due to unpublished null or negative findings. Future reviews with larger evidence bases may provide more definitive conclusions regarding publication bias.

## 5 Conclusion

To the best of our knowledge, this is the most comprehensive systematic review and meta-analysis to focus on the association between social capital and diabetes control. This comprehensive examination of the available evidence suggests a positive association between social capital and DM control despite various measurements, thus compensating for the shortcomings of existing reviews.

The present findings highlight the need for researchers to develop a robust theoretical framework that fully articulates the mechanisms through which social capital works to effect diabetes control, the various circumstances in which this occurs and how different components of social capital interact and mediate outcomes. Understanding the mechanisms through which this can occur would support policy makers to embed social capital as an underpinning feature of public health policies and interventions.

## Data Availability

The original contributions presented in the study are included in the article/supplementary material, further inquiries can be directed to the corresponding author.
